# Does Residents’ Satisfaction with the Neighbourhood Environment Relate to Residents’ Self-Rated Health? Evidence from Beijing

**DOI:** 10.3390/ijerph16245051

**Published:** 2019-12-11

**Authors:** Yiyi Chen, Mark Stephens, Colin A. Jones

**Affiliations:** The Urban Institute, Heriot-Watt University, Edinburgh EH14 4AS, UK; m.stephens@hw.ac.uk (M.S.); c.a.jones@hw.ac.uk (C.A.J.)

**Keywords:** self-rated health, neighbourhood green space, community green space

## Abstract

Objective: The aim of this study is to examine the association between satisfaction with two types of green space and residents’ self-rated health by comparing neighbourhood green space (NGS) and community green space (CGS) across spatial dimensions. Method: This study was based on 4291 workers from a large-scale individual survey of inhabitants of Beijing city in 2013. Multilevel ordered logistic regression analysis was used to examine the associations between residents’ satisfaction with the two types of green spaces and residents’ self-rated health. Results: Residents who are more satisfied with NGS and CGS have higher odds of reporting good self-rated health outcomes. Such effects are more pronounced for residents living close to NGS and tend to decline non-linearly over space. Conclusion: Additional results quantify the differentiated effects on self-rated health between urban and suburban residents. The findings of this study suggest that the effects of residents’ satisfaction with different types of green space on health benefits should be taken into account in the land-use design of green space preservation and development policies.

## 1. Introduction

For decades, empirical evidence has found that the interaction with green space using objective measures is associated with better physical health [1,2], physical activity [3,4,5,6,7,8], mental health [9,10,11,12,13,14,15], stress [16,17], and self-reported and self-rated health [18,19,20,21,22]. There is also growing evidence indicating that perceived greenness also contributes to health [23]. For example, results from one Australian study indicate that perceived neighbourhood green space is positively associated with mental and physical health [9]. Other studies have examined a related relationship: the effects of the quality of (perceived) neighbourhood green space and health across different cities [24] and population groups [25], although results are mixed. These studies highlight the importance of considering subjective measures of green space quality that may contribute to people’s health status.

However, few studies have explored the association between satisfaction derived from the quality of green space and people’s health outcomes. The importance of considering satisfaction arising from green space and people’s mental health was demonstrated in a 4-year followed-up longitude cohort study in Bradford [26]. This study found that Asian children who are more satisfied with green space are also more significantly likely to have fewer behavioural difficulties, less internalizing behaviour, and greater positive behaviour within different buffers from the residence to green space. Similarly, a related study examined the association between people’s subjective perception of neighbourhood green space (NGS) quality and mental health by asking the question, “How satisfied are you with the quality of green environment, using a five-point range Likert scale as the measurement?”. The study found only weak evidence of an association between satisfaction of NGS and mental health [27]. In addition to health benefits, another study explored the relationship between green space and fear of crime in New Zealand, indicating that residents who are satisfied with the quality of green space were more likely to report higher levels of life satisfaction compared to residents who are not satisfied [28]. However, these studies failed to differentiate between different types of green space that might play a moderating role in affecting the association between satisfaction with green space and health benefits.

This study is based on Beijing. Rapid urbanization has played an essential role in transforming the spatial pattern of urban land use in the city, including the spatial distribution of urban green space. However, as in other cities urban green space is not always equitably distributed [29]. In Beijing, green spaces are largely segregated by gated communities [30]. Many public parks are located in the urban area and some of them have been taken over and turned into community gardens or golf courses by developers. NGS such as woodlands and parks with good neighbourhood accessibility are located in the urban area within Third Ring Road. In contrast, fewer community green spaces (CGSs) and golf courses that people cannot freely access are located in the suburban area (beyond Second Ring Road) [31]. Such environmental inequality caused by spatial heterogeneity increases the difference in residents’ satisfaction with NGS and CGS, which results in variations in residents’ health outcomes. One underlying mechanism is that the effects of satisfaction with NGS and CGS may not be distributed equally across the space, which is in line with a recent study which suggests that access to green space and wellbeing may vary across distance buffers [32]. Further investigation is warranted because few studies have examined the heterogeneous spatial effects of satisfaction with green space on health by comparing NGS and CGS. To undertake such an analysis the research distinguishes between suburban and urban areas in Beijing. These are defined by reference to the city’s six ring roads as explained below.

Therefore, this study aims to fill in these gaps, as follows. First, following a previous paper [33], we decompose the perception of neighbourhood environment into two dimensions: satisfaction with NGS and satisfaction with CGS. We examine the association between socioeconomic characteristics and satisfaction with NGS and CGS, and then explore the association between residents’ satisfaction with NGS and CGS and residents self-rated health outcomes, adjusting for socioeconomic and neighbourhood characteristics. Thirdly, we conduct distance margins to examine whether the effects of residents’ satisfaction with NGS and CGS on health would decay in a linear manner or not. Finally, we examine whether residents whether living in the urban or suburban area moderates the association between satisfaction with NGS, CGS, and residents’ self-rated health outcomes.

## 2. Materials and Methods

### 2.1. Study Area

**Figure 1:**
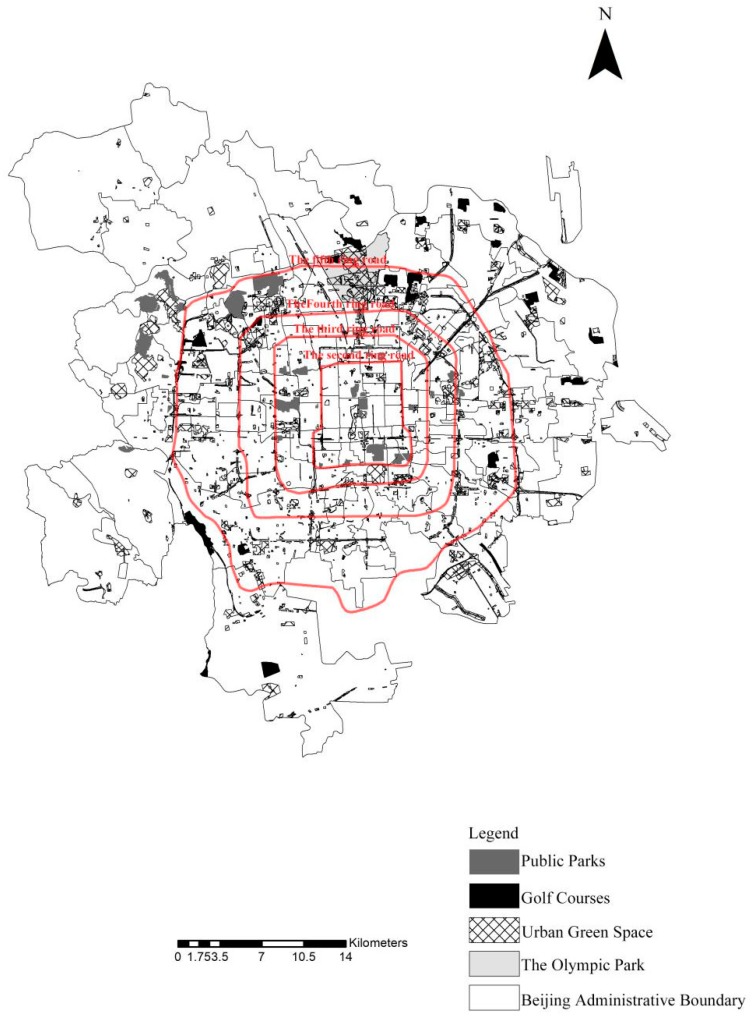
The location of the study area in Beijing.

Beijing, the capital of China, is one of the biggest megacities located at the northern tip of the roughly triangular North China Plain between 39°28′ and 41°05′ N and 115°25′ and 117°30′ E. The road network in Beijing consists of ring roads and radial roads. The road around the Forbidden City is identified as the First Ring Road, whilst the ring roads beyond this area represent the second, third, fourth, fifth, and sixth rings, and are defined by measuring the radial distance of each road from the city centre. The study area lies within the Fifth Ring Road of Beijing City (Figure 1). Four ring roads are arranged from the urban area to the suburban area, namely the Second, Third, Fourth, and Fifth Ring Roads. Third Ring Road is treated as the boundary between the urban and suburban areas.

### 2.2. Data Collection

Our analysis is based on a large-scale survey to examine the association between the satisfaction with NGS and CGS and residents’ self-rated health outcomes. The survey was undertaken by the Institute of Geographical Sciences and Natural Resources Research, Chinese Academy of Sciences in 2013 metropolitan Beijing. The questionnaires were posted to urban residents in proportion to the local population at individual, sub-district (*jiedao*), and district levels. The 2013 individual survey conducted a stratified proportional-to-population size sampling design, with about 7000 questionnaires circulated to metropolitan Beijing areas. All respondents were aged over 16. The survey collected personal information as well as data on perception of neighbourhood environment, self-reported subjective wellbeing, and satisfaction with income level. After the data cleaning process, 4291 sample respondents were included in the analysis. A two-level individual structure was formed based on residence locational information. Individual data were first assigned to *jiedao* and then nested into “subdistrict”. Some 16 districts and 134 *jiedao* were included in this study.

### 2.3. Self-Rated Health

We examined one health outcome in this study based on the question: “How do you rate your health condition?”. This question has been used as one of the most frequently used health indicators since the 1950s [34,35]. In contrast to physical ratings of actual and objective health, self-rated health tends to measure individuals’ perceptions and subjective health [34]. Responses to this question are measured using a five-point Likert-based scale ranging from “very bad” (1) to “very good” (5). This approach follows the WHO (1996) which suggests the following response scale: “very good”, “good”, “fair”, “bad”, and “very bad”. This scale is preferable to other measurements since it reflects subjective evaluations of health benefits at a scaled level. Regarding green space and self-rated health, studies have conducted self-rated health as predictive health indicator in investigating the association between greenness and residents’ health outcomes in previous studies [20,21].

### 2.4. Independent Variables

In terms of the dependent variables, perceived qualities of green space were treated as our key variables of interest. They include two dimensions: satisfaction with NGS and satisfaction with CGS. Both dimensions were measured by using the five-point like Likert-based scale ranging from “1 (perceived very weakly)” to “5 (perceived very strongly)”. For example, satisfaction with NGS and CGS was measured by a single item, “how well are you satisfied with the quality of your NGS and CGS as a whole?” Respondents answered on a 5-point Likert-based scale (1 = very dissatisfied, 2 = dissatisfied, 3 = neutral, 4 = satisfied, 5 = very satisfied). This measurement is feasible since it has been used in previous studies as a relative measure in evaluating perceptions of green space on wellbeing outcomes [26,36].

Respondents’ residential locations were recorded by geographic information system (GIS) coordinates. We further measured the straight-line distance in metres from each residential location to different social amenities. We then controlled for neighbourhood characteristics such as access to parks, hospitals, expressways, subway stations, and the central business district that may play an important role in influencing residents’ self-rated health outcomes [33]. It is worth noting that both subjective measurements (emotional aspects) and objective measurements (tangible aspects) were included in this study since they may capture different aspects of greenness that contribute to health differently [37].

### 2.5. Covariates

Regarding the covariates, we controlled for a set of individual characteristics that may play a significant role in moderating residents’ self-rated health outcomes, including residents’ homeownership, household registration system (*hukou*), age, gender, marital status, educational attainment level, employment status, income level, population density, and mobility. This adjustment is feasible and in line with previous studies that provided a review of the association between social-demographics and subjective wellbeing in large surveys [33]. In this study, we first identified whether residents were homeowners (1 = yes, 0 = no) and had local *hukou* registration (1 = yes, 0 = no). Of note, *hukou* registration was used as a criterion to distinguish between the urban and rural population [38]. We further determined residents’ educational attainment levels. We treated part-time employees as the base category in terms of employment status. We set residents whose monthly earnings exceeded 10,000 yuan as an income threshold for the comparison between residents with low income level and high-income level according to per capita monthly income of Beijing 2013. We included a categorical measure of age. Additionally, residents were asked to state “whether they have experienced residential relocation (i.e., moved) over the last five years”. We treated residents as movers if they had moved their residential location in the last five years, whereas non-movers were set as the reference category.

### 2.6. Statistical Analysis

The main strength of this study is the use of a multilevel ordered logistic model to examine the association between residents’ satisfaction with NGS and CGS and residents’ self-rated health, since our large-scale survey follows a hierarchical data structure (e.g., residents nested in subdistrict *jiedao* level and district levels) and the dependent variable is ordinal. The equation can be written as:

(1) $$\begin{array}{r} \eta_{cijk}=log\left\{Pr\left(y_{ijk}>C\right)\right\}=log\left(\frac{\mathrm{Pr}\left(y_{ijk}\leq c\right)}{1-\mathrm{Pr}\left(y_{ijk}\leq c\right)}\right)\\ =\beta_{0}+\beta_{1}X_{ijk}+\beta_{2}P_{nijk}+\nu_{k}+e_{jk} \end{array}$$

In this equation, $\eta_{cijk}$ represents the ordered logit prediction for the Cth cumulative comparison for the ith individual in the jth district of kth *jiedao*. $y_{ijk}$ is the odds of individual i in district j of *jiedao* k choosing the option in the five-point Likert scale. $X_{ijk}$ refers to the vector of individual-level control variables. $\beta_{0}$ refers to intercept of self-rated health in district level j of *jiedao* level k. $\beta_{1}$ and $\beta_{2}$ denote the corresponding coefficients.

In this study, the independent variable $P_{nijk}$ refers to three different characteristics. $P_{1\mathrm{ijk}}$ refers to perceived quality of green space, which is our key variable of interest. $P_{2\mathrm{ijk}}$ refers to the set of socioeconomic characteristics, $P_{3\mathrm{ijk}}$ refers to the neighbourhood characteristics. Of note, here $\beta_{2}$ refers to $\eta_{2}$, $\gamma_{3}$, and $\theta_{4}$. $y_{ijk}^{*}$ is written as: $\ln\frac{P_{ijk}}{1-p_{ijk}}$, with $y_{ijk}^{*}$ taking on the value of 1 with conditional probability $P_{ijk}$. Therefore, the equation can be transformed as:

(2) $$\begin{array}{cc} log\left(\frac{\mathrm{Pr}\left(y_{ijk}\leq c\right)}{1-\mathrm{Pr}\left(y_{ijk}\leq c\right)}\right) & =\beta_{0jk}+\beta_{1}X_{ijk}+\eta_{2}Preceived\;neighbourhood_{ijk}+\gamma_{3}Socioeconomic_{ijk}\\ & +\theta_{4}Neighbourhood_{ijk}+\nu_{k}+e_{jk} \end{array}$$

All analysis was performed using Stata, version 15 for Mac. We computed odds ratios (OR) with confidence inteval (CI) to present the estimation instead of using regression coefficients since the parameters for the multilevel logistic model can be hard to interpret.

## 3. Results

### 3.1. Descriptive Analysis

Table 1 presents the descriptive characteristics for the sample (*n* = 4291). Overall, 73% residents were satisfied and strongly satisfied with their self-rated health outcomes, followed by 23.9% who rated self-rated health as neutral; only a small proportion of residents rated themselves unsatisfied (2.8%) or very unsatisfied with their self-rated health outcomes (0.3%). In terms of satisfaction with neighbourhood characteristics, 55.9% residents reported they were satisfied and strongly satisfied with NGS compared to 13.3% which were dissatisfied and strongly dissatisfied. Forty-nine percent of residents were satisfied or strongly satisfied with CGS compared to 13.3% dissatisfied and strongly dissatisfied. Regarding their socioeconomic characteristics, nearly 40% respondents were young (aged between 20 and 29). It was found that 61.5% residents were married and 63.9% had attained higher educational level. In total, 37.6% residents monthly earned over 10,000 yuan and 24.9% of residents had moved in the last five years.

To test the potential collinearity between perception characteristics and economic demographics characteristics and neighbourhood characteristics in the estimated model, we applied the variation inflation factor (VIF) diagnostics in Stata. The results show that none of the VIFs are greater than 3, which indicates that there are no serious collinearity issues in the estimation model. We therefore kept all the variables in the multilevel model as reported in Table 2.

### 3.2. Baseline Results

Table 2 reports the baseline results by conducting three columns. Column 1 and 2 examine the association between socioeconomic characteristics and residents’ satisfaction with NGS and CGS respectively. Column 3 examines the association between satisfaction with NGS and CGS and self-rated health outcome. Socioeconomic characteristics and neighbourhood characteristics are further adjusted in column 4.

Results from column 1 and 2 suggest that residents with higher educational attainment level are more likely to have a higher score of satisfaction with both NGS and CGS. Notably, residents who owned a house tended to report higher score of satisfaction with CGS. The result from column 3 indicates that residents who are more satisfied with NGS and CGS have higher odds to report good self-rated health outcomes compared to those reports unsatisfied with NGS and CGS. Such effects remained significant and robust after adjustment for socioeconomic and neighbourhood characteristics. Specifically, residents who were more satisfied with NGS were 24.6% more likely to report extremely good health outcomes (self-rated health score of 5 compared to residents who were more satisfied with community green space, accounting for 9.7%).

Regarding socioeconomic characteristics, residents who had a higher income level tended to have higher odds of reporting good health outcomes, whereas *hukou* status, home ownership, sex, marital status educational attainment level, mobility, and population density were not significantly associated with residents’ self-rated health outcomes. Additionally, elderly residents were 25% less likely to report being good self-rated health outcomes compared to younger residents. Full-time employees had lower odds of being healthy than part-time employees.

Regarding neighbourhood characteristics, those with residential proximity to central business districts and hospitals were 3.2% and 16.8% more likely to be in good health than those living far from parks and expressways. Conversely, residential proximity to parks and expressways was negatively associated with the odds of reporting higher self-rated health outcomes, accounting for 3.5% and 4% respectively.

### 3.3. Spatial Heterogeneity across Distance Buffers

Table 3 explores the heterogeneity base on our baseline estimates in different distance buffer thresholds. Columns 1 to 6 reports the results by including residents living at different distance intervals from residence to urban greenness. We chose a 500-m buffer as the base category since 500 m is treated as a walkable distance margin from residence to green space [39].

Results from Table 3 indicate that residents who are more satisfied with NGS and living close to urban greenness have the highest odds of reporting good self-rated health outcomes. Such effects tend to decline with distance away from urban greenness in a non-linear manner. Specifically, residents who were more satisfied with NGS were more likely to report having good self-rated health outcomes if living within a 500-m margin of their residence compared with residents living between 500 and 1000 m and more than 2000 m from urban greenness. Interestingly, such decay tended to reverse with respect to residents living within 2000–3000 m margins from their place of residence to urban greenness and decline again among residents living beyond 4000 m. Regarding residents’ satisfaction with CGS, no significant correlation of residents’ self-rated outcomes was found with regard to residents who lived in 500–4000 m distance margins. However, residents tended to have higher odds of reporting being good self-rated health outcomes if they lived beyond 4000 m from their place of residence to urban greenness. One underlying mechanism is that most urban green space in Chinese communities is private, and most people cannot access them because most Chinese communities are gated. Therefore, residents are more likely to perceive better health outcomes due to access to NGSs such as public parks and forests.

### 3.4. Differences between Urban and Suburban

Table 4 summarises the spatial variations in the association between satisfaction with NGS and CGS and residents’ self-rated health outcomes for residents living in the urban and suburban area respectively.

Table 4 reports that urban residents (residents living in the urban area) who are more satisfied with NGS are 26% more likely to report being good (self-rated) health outcomes compared to suburban residents (reported 23%). Conversely, in terms of the satisfaction with CGS, suburban residents were 14.4% more likely to report having good self-rated health outcomes compared to urban residents (reported 3.4%). These results indicate that urban residents who were more satisfied with NGS were more likely to report having good self-rated health outcomes compared to suburban residents who were more satisfied with CGS. On the contrary, suburban residents who were more satisfied with CGS tended to report being in good health compared to urban residents who were more satisfied with NGS.

## 4. Discussion

This paper not only examines the relationship between socioeconomic characteristics and residents’ satisfaction with NGS and CGS, but also compares the differences in health disparities between residents’ satisfaction with NGS and CGS, adjusting for socioeconomic and neighbourhood characteristics. It further examines the role of the spatial dimension of satisfaction with NGS and CGS in moderating residents’ self-rated health outcome across the spatial dimension.

### 4.1. The Effect of Satisfaction with Neighbourhood Green Space (NGS) and Community Green Space (CGS) on Health

Our findings suggest that residents with a higher educational attainment level tend to have a higher satisfaction score with both NGS and CGS. This finding is consistent with results with respect to agreement between objective and subjective measures of green space [23]. As expected, residents’ levels of satisfaction with NGS and CGS are associated with better self-rated health outcomes. Such effects remain stable and robust after adjusting for socio-economic and neighbourhood characteristics, which is consistent with previous findings that satisfaction with green space could promote residents’ health benefits [26]. This is plausible, since greater satisfaction with the environment would promote individuals’ awareness to use and access urban green space and that potentially enhances people’s health [40].

### 4.2. Satisfaction with Neighbourhood Green Space (NGS) and Community Green Space (CGS) on Health across Spatial Dimensions

The association between satisfaction with NGS and CGS tends to decline with distance from green spaces in a non-linear matter. Specifically, residents who are more satisfied with NGS and living close to urban greenness have the highest odds of reporting good health. Conversely, residents who are more satisfied with CGS are more likely to report better self-rated health levels and are living beyond 4000 m margins from residence to urban greenness. One possible explanation of this result is that CGS plays an essential role in promoting residents’ health level in an area where accessible NGS is limited.

A comparison of the model specifications for urban and suburban residents suggests that residential location significantly moderates the association between satisfaction with NGS and CGS and residents’ self-rated health outcomes. The results suggest that urban residents who are more satisfied with NGS tend to report good self-rated health outcomes compared to residents living in suburban areas. Interestingly, such disparities depend on residential location with residents living in the urban area more sensitive to NGS while residents living in the suburban area are more sensitive to CGS. One underlying mechanism of this rationale is that the lack of NGS in the suburban area encourages residents to access to community green space, even though some community green spaces are not accessible (e.g., private garden). This finding is in line with one study suggesting that fewer green spaces are located in the edge of the city, but perform a similar function as urban parks and forest so potentially contribute to people’s health benefits [41].

### 4.3. Contributions and Limitations

These results suggest that local government and urban designers might not need to design new public parks in suburban areas. For one thing, governments can maintain some communities with a high quality of community green space that providing enough aesthetic greenness and attract more residents to access it. For another, green space in different communities should be connected to surrounding CGS or NGS in order to provide residents with more easily accessible green space. This is feasible, since the Chinese central government has issued a new urban planning directive that gated communities will gradually be opened to the public including the communities’ facilities and greenness, which promotes the integration between NGS and CGS [42].

Studies in previous decades have witnessed the rise of the “Healthy Cities” movement [43] and the policy implications towards promoting environmental justice [29]. To place this movement in urban contexts, the contribution of neighbourhood environment on subjective residents’ health is substantial [33]. Notably, most urban land in China is owned and controlled by local government and land leasing has become the easiest way for local government to raise money [44]. Local authorities are eager to expand urban areas rapidly, where less land is allocated for public green space. Furthermore, a large number of wild green spaces in the suburban areas (e.g., public green space, forest parks, woodland) have been requisitioned for real estate development, which exacerbates environment inequalities [45]. In this study, comparisons are made between perceived health benefits associated with residents living in urban and suburban areas. The results highlight the significant influence on residential location on the difference of perception of NGS and CGS on residents’ health disparities. This is an important finding, suggesting that governments and developers should give priority to maintaining and beautifying community green space instead of constructing new public parks in suburban areas. In this sense, site selection should not only consider the potential benefits arising from the land value, but also should consider the underlying factors that affect residents’ health and wellbeing.

Additionally, limitations to this study should be noted. First, we use subjective assessments to measure individuals’ satisfaction with green space and health status, which could lead to self-selection bias in the estimation results. For example, respondents who have higher incomes may choose to live close to green space and are more likely to experience better self-rated health outcomes than middle- or low-income residents. In this case, our baseline model will underestimate the effects of perception of neighbourhood environment on residents’ self-rated health outcomes. Second, since our dataset is designed in the form of a cross-sectional survey, we are therefore not able to avoid the causal effects that may have led to a bias in our estimations. Third, human behaviour in urban contexts such as physical exercise that is not recorded in this survey will affect people’s health expectations and preferences in the longer term [46]. For example, individuals who smoke or live other unhealthy lifestyles may generate interaction effects with the perception of neighbourhood environment on health. These issues warrant further studies. More research on residents’ use of public greenness on wellbeing are needed to understand the role that that dynamic residential experiences and social cohesion play in subjective perceptions [47]. This also warrants further investigation. Despite these limitations, our findings provide new insights on the spatially heterogeneous roles of perception of neighbourhood environment in shaping self-rated health patterns across spatial dimensions. More research is needed to understand the precise underlying mechanisms and urban policies that could facilitate healthy lifestyles in megacities.

## 5. Conclusions

The overall finding from this study indicates that satisfaction with NGS and CGS is positively associated with residents’ self-rated health outcomes. These effects are more pronounced for residents living close to NGSs and tend to decline in a non-linear manner over space, with residents who are more satisfied with NGSs being more sensitive to greenness located beyond 4000 m from their home. Residential location significantly moderates the relationship between satisfaction with NGS and CGS and residents’ self-rated health outcomes. Urban residents who are more satisfied with NGS are more likely to report better self-rated health outcomes compared to suburban residents. These results highlight the importance of considering subjective assessment of green space quality with respect to health benefits, providing potential advice for stakeholders to reconsider the environment justice in the provision of unnecessary green space in urban design.

## Figures and Tables

**Table 1:** Statistical description.

Variable	Description	*n*	Proportion (%)
Self-rated health (SRH)		4291	
	Extremely poor health	13	0.3
	Poor health	119	2.8
	Neutral	1027	23.9
	Good health	2348	54.7
	Extremely good health	784	18.3
Satisfaction with NGS			
	Strongly dissatisfied with NGS	27	0.6
	Dissatisfied with NGS	369	8.6
	Neutral	1497	34.9
	Satisfied with NGS	2036	47.5
	Strongly satisfied with NGS	362	8.4
Satisfaction with CGS			
	Strongly dissatisfied with CGS	55	1.3
	Dissatisfied with CGS	515	12.0
	Neutral	1622	37.8
	Satisfied with CGS	1779	41.5
	Strongly satisfied with CGS	320	7.5
SES			
*Hukou*	(People without *hukou* registration as reference category)	2907	67.8
Homeownership	(Non-homeowner as reference category)	1477	34.4
Age			
	20–29	1702	39.7
	30–39	1273	29.7
	40–49	693	16.2
	50–59	375	8.7
	60+	132	3.1
Sex	(Female as the reference category)	2170	50.6
Marital status	(Unmarried as the reference category)	2639	61.5
Educational level			
	Junior high school or below (reference category)	407	9.5
	High school	1142	26.6
	University or College	2369	55.2
	Master’s or above	373	8.7
Employment status	(Part-time employment as the reference category)	3260	84.4
Income level	(Monthly earnings of ≤10,000 yuan as the reference category)	1613	37.6
Mobility	(Non-mover as the reference category)	1067	24.9
Population density	Density of population in study area	4291	12.5
Neighbourhood variables			
	Distance to the park (km)	4291	3.3
	Distance to the central business district (km)	4291	10.6
	Distance to the hospital (km)	4291	0.5
	Distance to the expressway (km)	4291	3.5
	Distance to the subway (km)	4291	1.9

Notes: We used the mean to present variables including population density, the metric distance to the park, the metric distance to central business district, the metric distance to the hospital, the metric distance to the expressway, and the metric distance to the subway. CGS: community green space; NGS: neighbourhood green space; SES: socioeconomic status.

**Table 2:** Odds ratios with 95% confidence intervals predicting residents’ satisfaction with NGS and CGS and self-rated health level. OR: odds ratio. CI: confidence inteval.

	Model 1 OR/(95% CI)	Model 2 OR/(95% CI)	Model 3 OR/(95% CI)	Model 4 OR/(95% CI)
Satisfaction with NGS			1.254 ***	1.246 ***
			[1.142, 1.378]	[1.133, 1.369]
Satisfaction with CGS			1.119 **	1.094 **
			[1.025, 1.221]	[1.001, 1.195]
Hukou	0.973	0.910		1.094
	[0.845, 1.121]	[0.792, 1.046]		[0.948, 1.262]
Homeownership	1.070	1.131 *		1.013
	[0.927, 1.234]	[0.984, 1.300]		[0.878, 1.170]
Age	0.946	0.966		0.747 ***
	[0.882, 1.014]	[0.902, 1.034]		[0.696, 0.803]
Sex	0.952	0.927		0.907
	[0.840, 1.077]	[0.821, 1.047]		[0.800, 1.028]
Marital status	0.979	1.067		0.936
	[0.854, 1.123]	[0.932, 1.221]		[0.814, 1.077]
Junior high school and below	1.000	1.000		1.000
	[1.000, 1.000]	[1.000, 1.000]		[1.000, 1.000]
High school	0.884	1.029		0.999
	[0.706, 1.108]	[0.825, 1.283]		[0.794, 1.257]
University or College	1.022	1.172		1.173
	[0.817, 1.280]	[0.940, 1.462]		[0.932, 1.478]
Master or above	1.343 *	1.355 **		1.190
	[0.995, 1.813]	[1.012, 1.814]		[0.876, 1.616]
Employment status	0.977	1.037		0.839 **
	[0.822, 1.160]	[0.875, 1.228]		[0.704, 1.000]
Income (>10,000 yuan)	1.044	1.058		1.511 ***
	[0.919, 1.185]	[0.933, 1.198]		[1.327, 1.720]
Mobility	0.935	0.925		0.979
	[0.810, 1.080]	[0.803, 1.066]		[0.845, 1.135]
Population density	0.999	1.004		1.000
	[0.994, 1.004]	[0.999, 1.008]		[0.994, 1.006]
Distance to park				0.965 **
				[0.932, 1.000]
Distance to central business district				1.032 ***
				[1.017, 1.048]
Distance to hospital				1.168 *
				[0.974, 1.400]
Distance to expressway				0.960 **
				[0.930, 0.992]
Distance to subway				1.008
				[0.965, 1.052]
Thresholds for cumulative logit				
First	0.004 ***	0.011 ***	0.010 ***	0.004 ***
	[0.002, 0.009]	[0.005, 0.023]	[0.005, 0.019]	[0.002, 0.010]
Second	0.070 ***	0.136 ***	0.103 ***	0.043 ***
	[0.035, 0.141]	[0.069, 0.268]	[0.069, 0.153]	[0.019, 0.096]
Third	0.626	1.042	1.261	0.551
	[0.314, 1.248]	[0.531,2.044]	[0.873, 1.821]	[0.249, 1.219]
Fourth	10.749 ***	14.441 ***	17.059 ***	8.159 ***
	[5.366,21.532]	[7.309,28.532]	[11.682,24.912]	[3.681, 18.084]
Variance in district level	1.457 **	1.307 **	1.560 **	1.533 **
	[1.023, 2.075]	[1.006, 1.700]	[1.102, 2.209]	[1.068, 2.199]
Variance in *jiedao* level	1.480 **	1.261 ***	1.049	1.077
	[1.098, 1.997]	[1.066, 1.491]	[0.972, 1.131]	[0.974, 1.190]
*n*	4283.000	4283.000	4283.000	4283.000
AIC	9725.596	1.0 × 10 ^4^	9272.572	9118.103
BIC	9840.120	1.0 × 10 ^4^	9323.471	9277.163
chi^2^	24.370	32.695	61.605	243.890
*p*	0.018	0.001	0.000	0.000

Notes: Model 1 explores the association between socioeconomic characteristics and residents’ satisfaction with NGS. Model 2 examines the association between socioeconomic characteristics and residents’ satisfaction with CGS. Model 3 analyses the relationship between satisfaction with NGS and CGS and self-rated health with no adjustments. Model 4 additionally adjusts for socioeconomic characteristics and neighbourhood characteristics. Exponentiated coefficients; standard errors in parentheses; * *p* < 0.10, ** *p* < 0.05, *** *p* < 0.01; CGS: community green space; NGS: neighbourhood green space; AIC: Akaike information criterion; BIC: Bayesian information criterion.

**Table 3:** Odds ratios with 95% confidence intervals predicting residents’ self-rated health level across different distance intervals from residence to urban green space.

	<500 m OR/(95% CI)	500–1000 m OR/(95% CI)	1000–2000 m OR/(95% CI)	2000–3000 m OR/(95% CI)	3000–4000 m OR/(95% CI)	>4000 m OR/(95% CI)
Satisfaction with NGS	1.629 *	1.160	1.116	1.393 ***	1.629 ***	1.187 *
	[0.959, 2.765]	[0.843, 1.595]	[0.936, 1.330]	[1.098, 1.767]	[1.248, 2.127]	[0.987, 1.428]
Satisfaction with CGS	1.005	0.924	1.042	1.165	0.932	1.344 ***
	[0.589, 1.713]	[0.686, 1.245]	[0.889, 1.222]	[0.927, 1.464]	[0.725, 1.198]	[1.130, 1.598]
*Hukou*	1.440	1.098	0.845	1.285	0.911	1.329 **
	[0.612,3.387]	[0.676, 1.782]	[0.630, 1.132]	[0.888, 1.860]	[0.623, 1.334]	[1.026, 1.721]
Homeownership	1.026	0.768	1.101	0.906	1.126	1.029
	[0.472, 2.230]	[0.499, 1.181]	[0.838, 1.447]	[0.628, 1.309]	[0.749, 1.691]	[0.774, 1.368]
Age	0.757	0.601 ***	0.744 ***	0.846 *	0.537 ***	0.837 **
	[0.522, 1.098]	[0.482, 0.749]	[0.652, 0.850]	[0.707, 1.012]	[0.434, 0.665]	[0.724, 0.969]
Sex	1.302	0.822	0.875	1.028	0.979	0.849
	[0.640, 2.649]	[0.560, 1.207]	[0.690, 1.110]	[0.745, 1.419]	[0.689, 1.391]	[0.666, 1.083]
Marital status	0.884	0.720	1.072	1.052	0.605**	1.026
	[0.476, 1.643]	[0.472, 1.099]	[0.815, 1.410]	[0.763, 1.450]	[0.398, 0.920]	[0.765, 1.376]
Junior high school and below	1.000	1.000	1.000	1.000	1.000	1.000
	[1.000, 1.000]	[1.000, 1.000]	[1.000, 1.000]	[1.000, 1.000]	[1.000, 1.000]	[1.000, 1.000]
High school	1.046	0.878	0.820	1.463	0.715	1.130
	[0.265,4.119]	[0.411, 1.874]	[0.501, 1.341]	[0.847, 2.526]	[0.379, 1.348]	[0.740, 1.727]
University or College	1.213	1.021	1.023	2.228 ***	0.705	1.096
	[0.304,4.846]	[0.471, 2.212]	[0.625, 1.675]	[1.269,3.910]	[0.374, 1.329]	[0.717, 1.677]
Master or above	0.372	0.817	1.250	2.161 **	1.062	0.881
	[0.066, 2.093]	[0.279, 2.388]	[0.673, 2.324]	[1.033,4.518]	[0.473, 2.387]	[0.485, 1.599]
Employment status	1.280	0.704	0.968	0.627 **	0.671	0.881
	[0.488,3.354]	[0.413, 1.200]	[0.695, 1.348]	[0.406, 0.969]	[0.403, 1.118]	[0.624, 1.243]
Income (>10,000 yuan)	1.531	1.494 *	1.171	1.832 ***	1.421 *	1.927 ***
	[0.658,3.566]	[0.996, 2.243]	[0.923, 1.486]	[1.320, 2.541]	[0.981, 2.056]	[1.482, 2.507]
Mobility	0.642	1.224	1.060	1.100	0.917	0.855
	[0.271, 1.518]	[0.743, 2.018]	[0.773, 1.454]	[0.754, 1.604]	[0.619, 1.358]	[0.661, 1.106]
Population density	1.096 ***	1.004	0.991 *	1.009	1.008	0.981
	[1.025, 1.172]	[0.989, 1.020]	[0.981, 1.001]	[0.996, 1.022]	[0.993, 1.024]	[0.937, 1.027]
Distance to park	0.019 ***	2.174	0.817	1.394	0.900	0.892 ***
	[0.001, 0.383]	[0.580,8.148]	[0.534, 1.251]	[0.819, 2.372]	[0.469, 1.728]	[0.820, 0.969]
Distance to central business district	1.142 ***	0.977	1.034 **	1.024	1.039	1.070 ***
	[1.059, 1.231]	[0.936, 1.020]	[1.003, 1.065]	[0.990, 1.060]	[0.985, 1.096]	[1.024, 1.119]
Distance to hospital	10.838 **	1.658	1.296	1.208	1.272	1.217 *
	[1.542,76.183]	[0.583,4.714]	[0.668, 2.518]	[0.587, 2.487]	[0.686, 2.359]	[0.964, 1.537]
Distance to expressway	0.882	1.196 **	0.959	0.978	0.868 **	0.947 *
	[0.640, 1.215]	[1.038, 1.379]	[0.881, 1.045]	[0.876, 1.091]	[0.776, 0.971]	[0.894, 1.003]
Distance to subway	0.961	0.805	0.869	1.129	1.006	1.013
	[0.489, 1.889]	[0.609, 1.063]	[0.728, 1.037]	[0.949, 1.344]	[0.858, 1.179]	[0.959, 1.071]
Thresholds for cumulative logit						
First	0.313	0.005 ***	0.001 ***	0.200	0.000 ***	0.011 ***
	[0.003,35.966]	[0.000, 0.081]	[0.000, 0.007]	[0.019, 2.143]	[0.000, 0.009]	[0.002, 0.068]
Second	5.608	0.123	0.018 ***	0.841	0.003 ***	0.113 ***
	[0.052,604.788]	[0.008, 1.844]	[0.003, 0.110]	[0.087,8.166]	[0.000, 0.065]	[0.024, 0.536]
Third	75.085 *	1.869	0.195 *	10.972 **	0.047 **	1.413
	[0.667,8455.775]	[0.126, 27.690]	[0.033, 1.142]	[1.145, 105.157]	[0.002, 0.995]	[0.306,6.529]
Fourth	1.000	1.059	1.088	1.002	1.051	1.595
	[1.000, 1.000]	[0.934, 1.201]	[0.810, 1.460]	[0.885, 1.136]	[0.881, 1.253]	[0.739,3.445]
Variance in district level	1.072	1.000	1.111	1.029	1.001	1.086
	[0.772, 1.487]	[1.000, 1.000]	[0.921, 1.342]	[0.912, 1.162]	[0.845, 1.187]	[0.853, 1.382]
Variance in *jiedao* level			2.848	161.854 ***	0.850	24.203 ***
			[0.486, 16.693]	[16.451, 1592.395]	[0.041, 17.802]	[5.177, 113.144]
*n*	146.000	457.000	1215.000	705.000	579.000	1181.000
AIC	344.732	970.092	2645.086	1547.506	1198.907	2514.828
BIC	413.355	1064.960	2772.648	1661.461	1307.940	2641.680
*p*	0.022	0.003	0.000	0.000	0.000	0.000

Exponentiated coefficients; 95% confidence intervals in brackets* *p* < 0.10, ** *p* < 0.05, *** *p* < 0.01; CGS: community green space; NGS: neighbourhood green space; AIC: Akaike information criterion; BIC: Bayesian information criterion.

**Table 4:** Odds ratios with 95% confidence intervals predicting residents’ self-rated health level between urban and suburban area.

	Urban OR/(95% CI)	Suburban OR/(95% CI)
Satisfaction with NGS	1.261 ***	1.230 ***
	[1.081, 1.470]	[1.088, 1.390]
Satisfaction with CGS	1.034	1.144 **
	[0.899, 1.191]	[1.019, 1.285]
*Hukou*	0.741 **	1.335 ***
	[0.581, 0.946]	[1.114, 1.601]
Homeownership	1.045	1.019
	[0.829, 1.317]	[0.846, 1.226]
Age	0.670 ***	0.794 ***
	[0.597, 0.752]	[0.723, 0.871]
Sex	0.920	0.875
	[0.752, 1.126]	[0.744, 1.029]
Marital status	0.921	0.936
	[0.739, 1.147]	[0.778, 1.126]
Junior high school and below	1.000	1.000
	[1.000, 1.000]	[1.000, 1.000]
High school	0.962	0.990
	[0.656, 1.412]	[0.740, 1.326]
University or College	1.021	1.216
	[0.691, 1.509]	[0.911, 1.625]
Master or above	1.036	1.199
	[0.619, 1.733]	[0.814, 1.766]
Employment status	0.755 *	0.898
	[0.561, 1.015]	[0.719, 1.121]
Income (>10, 000 yuan)	1.431 ***	1.609 ***
	[1.169, 1.753]	[1.354, 1.913]
Mobility	0.901	1.020
	[0.704, 1.154]	[0.847, 1.227]
Population density	0.999	1.002
	[0.991, 1.008]	[0.993, 1.011]
Distance to park	1.072	0.950 ***
	[0.946, 1.215]	[0.914, 0.987]
Distance to central business district	1.090 ***	1.022
	[1.031, 1.152]	[0.996, 1.050]
Distance to hospital	1.167	1.266 **
	[0.637, 2.137]	[1.041, 1.540]
Distance to expressway	1.092 *	0.956 **
	[0.998, 1.194]	[0.916, 0.998]
Distance to subway	0.749 ***	1.034
	[0.628, 0.893]	[0.987, 1.083]
Thresholds for cumulative logit		
First	0.002 ***	0.007 ***
	[0.000, 0.008]	[0.002, 0.024]
Second	0.019 ***	0.070 ***
	[0.005, 0.076]	[0.024, 0.204]
Third	0.229 **	0.981
	[0.058, 0.897]	[0.345, 2.792]
Fourth	3.476 *	15.030 ***
	[0.891, 13.558]	[5.255,42.987]
Variance in district level	1.123	1.660 **
	[0.777, 1.625]	[1.007, 2.738]
Variance in *jiedao* level	1.004	1.177 *
	[0.976, 1.033]	[0.992, 1.397]
*n*	1695.000	2588.000
AIC	3651.699	5471.585
BIC	3787.585	5618.051
chi^2^	130.457	145.427
*p*	0.000	0.000

Exponentiated coefficients; Standard errors in parentheses; * *p* < 0.10, ** *p* < 0.05, *** *p* < 0.01; CGS: community green space; NGS: neighbourhood green space; AIC: Akaike information criterion; BIC: Bayesian information criterion.
